# Supplementary material for: Multi‐dataset identification of innovative feature genes and molecular mechanisms in keratoconus
Source: J Cell Mol Med. 2024 Sep 19;28(18):e70079. doi: 10.1111/jcmm.70079 (PMC11412914; doi:10.1111/jcmm.70079)
Supplement: Supplementary file 2 — Material S2. [file JCMM-28-e70079-s002.zip › 7.GSEA analysis/2.ATOH7/KEGG_PANTOTHENATE_AND_COA_BIOSYNTHESIS.html]

Details for gene set KEGG\_PANTOTHENATE\_AND\_COA\_BIOSYNTHESIS[GSEA]

|  || Dataset | ATOH7.group.cls#h\_versus\_l |
| Phenotype | group.cls#h\_versus\_l |
| Upregulated in class | l |
| GeneSet | KEGG\_PANTOTHENATE\_AND\_COA\_BIOSYNTHESIS |
| Enrichment Score (ES) | -0.25972465 |
| Normalized Enrichment Score (NES) | -0.67747825 |
| Nominal p-value | 0.87341774 |
| FDR q-value | 1.0 |
| FWER p-Value | 1.0 |
Table: GSEA Results Summary

  

Fig 1: Enrichment plot: KEGG\_PANTOTHENATE\_AND\_COA\_BIOSYNTHESIS      
 Profile of the Running ES Score & Positions of GeneSet Members on the Rank Ordered List

  

| SYMBOL | TITLE | RANK IN GENE LIST | RANK METRIC SCORE | RUNNING ES | CORE ENRICHMENT || 1 | VNN2 | na | 5564 | 0.184 | -0.0481 | Yes |
| 2 | DPYD | na | 5776 | 0.177 | 0.0541 | Yes |
| 3 | ENPP1 | na | 6112 | 0.168 | 0.1473 | Yes |
| 4 | PANK4 | na | 7378 | 0.133 | 0.1923 | Yes |
| 5 | BCAT2 | na | 15795 | 0.014 | -0.0413 | No |
| 6 | DPYS | na | 16196 | 0.011 | -0.0459 | No |
| 7 | ENPP3 | na | 17063 | 0.004 | -0.0685 | No |
| 8 | COASY | na | 18678 | -0.010 | -0.1088 | No |
| 9 | PANK2 | na | 19145 | -0.014 | -0.1136 | No |
| 10 | PPCS | na | 22020 | -0.043 | -0.1700 | No |
| 11 | PANK3 | na | 22168 | -0.045 | -0.1469 | No |
| 12 | PANK1 | na | 22434 | -0.048 | -0.1254 | No |
| 13 | UPB1 | na | 25229 | -0.089 | -0.1513 | No |
| 14 | PPCDC | na | 26966 | -0.125 | -0.1253 | No |
| 15 | VNN1 | na | 31635 | -0.275 | -0.0918 | No |
| 16 | BCAT1 | na | 32042 | -0.296 | 0.0774 | No |
Table: GSEA details [plain text format]

  

Fig 2: KEGG\_PANTOTHENATE\_AND\_COA\_BIOSYNTHESIS      
 Blue-Pink O' Gram in the Space of the Analyzed GeneSet

  

Fig 3: KEGG\_PANTOTHENATE\_AND\_COA\_BIOSYNTHESIS: Random ES distribution      
 Gene set null distribution of ES for **KEGG\_PANTOTHENATE\_AND\_COA\_BIOSYNTHESIS**
